# Uncovering the Genetic Landscape for Multiple Sleep-Wake Traits

**DOI:** 10.1371/journal.pone.0005161

**Published:** 2009-04-10

**Authors:** Christopher J. Winrow, Deanna L. Williams, Andrew Kasarskis, Joshua Millstein, Aaron D. Laposky, He S. Yang, Karrie Mrazek, Lili Zhou, Joseph R. Owens, Daniel Radzicki, Fabian Preuss, Eric E. Schadt, Kazuhiro Shimomura, Martha H. Vitaterna, Chunsheng Zhang, Kenneth S. Koblan, John J. Renger, Fred W. Turek

**Affiliations:** 1 Department of Depression and Circadian Rhythms, Merck Research Laboratories, West Point, Pennsylvania, United States of America; 2 Center for Sleep and Circadian Biology, Northwestern University, Evanston, Illinois, United States of America; 3 Genetics Department, Rosetta Inpharmatics LLC, a wholly owned subsidiary of Merck & Co., Inc., Seattle, Washington, United States of America; 4 Informatics Custom Analysis Department, Rosetta Inpharmatics LLC, a wholly owned subsidiary of Merck & Co., Inc., Seattle, Washington, United States of America; Duke University, United States of America

## Abstract

Despite decades of research in defining sleep-wake properties in mammals, little is known about the nature or identity of genes that regulate sleep, a fundamental behaviour that in humans occupies about one-third of the entire lifespan. While genome-wide association studies in humans and quantitative trait loci (QTL) analyses in mice have identified candidate genes for an increasing number of complex traits and genetic diseases, the resources and time-consuming process necessary for obtaining detailed quantitative data have made sleep seemingly intractable to similar large-scale genomic approaches. Here we describe analysis of 20 sleep-wake traits from 269 mice from a genetically segregating population that reveals 52 significant QTL representing a minimum of 20 genomic loci. While many (28) QTL affected a particular sleep-wake trait (e.g., amount of wake) across the full 24-hr day, other loci only affected a trait in the light or dark period while some loci had opposite effects on the trait during the light vs. dark. Analysis of a dataset for multiple sleep-wake traits led to previously undetected interactions (including the differential genetic control of number and duration of REM bouts), as well as possible shared genetic regulatory mechanisms for seemingly different unrelated sleep-wake traits (e.g., number of arousals and REM latency). Construction of a Bayesian network for sleep-wake traits and loci led to the identification of sub-networks of linkage not detectable in smaller data sets or limited single-trait analyses. For example, the network analyses revealed a novel chain of causal relationships between the chromosome 17@29cM QTL, total amount of wake, and duration of wake bouts in both light and dark periods that implies a mechanism whereby overall sleep need, mediated by this locus, in turn determines the length of each wake bout. Taken together, the present results reveal a complex genetic landscape underlying multiple sleep-wake traits and emphasize the need for a systems biology approach for elucidating the full extent of the genetic regulatory mechanisms of this complex and universal behavior.

## Introduction

The behavioral states of sleep and wake, as defined by electroencephalogram (EEG) and electromyogram (EMG) activity, are composed of multiple sub-component measures with sleep itself being divided into the primary states of Rapid Eye Movement (REM) and Non Rapid Eye Movement (NREM) sleep in mammals [1], [2]. Although there is considerable evidence supporting a strong genetic basis for some sleep-wake traits and sleep disorders [3], as well as speculation on the polygenic nature of sleep due to the complexity of the behavior [4], little has been done to unravel the complex network of genetic and physiological interactions that must underlie this universal behavior in mammals. While sleep-wake recordings in recombinant mouse strains have identified a limited number of significant or “suggestive” quantitative trait loci (QTL) for a few sleep-wake measurements [5], [6], [7], and a small number of genes in these QTL have been found to be associated with some individual sleep-wake properties [8], [9], no previous attempts have been made to record sleep in a large genetically segregating population of mice in order to utilize modern genetic and genomic approaches to study sleep. As a first step to understand the full genetic complexity (i.e., the genetic landscape) underlying the regulation of sleep, we carried out a genome wide scan for the various components of this complex mammalian behavior by examining linkage between 2,310 informative single nucleotide polymorphisms (SNPs) and 20 sleep-wake traits in 269 male mice from a genetically segregating population. In addition, we examined the relationships among the different traits to assess whether sleep-wake traits that have been presumed to be related actually share common genetic influences.

The breeding scheme to produce a segregating mouse population was set up to enable both the identification of QTL, as well as to allow for the mapping of a chemically-induced unknown mutation that resulted in a greater amount of wakefulness (referred to as the *Sleepless* mutation) on a C57BL/6J (B6) background. To increase genetic variants for QTL analysis, we selected a counter strain that had substantially different sleep-wake characteristics from B6; the BALB/cByJ (BALB) strain showed significant differences in sleep fragmentation (e.g. more stage shifts, shorter sleep/wake bout durations) but similar amounts of wake compared to wild-type B6 mice. The *Sleepless* mutation segregates as a single autosomal dominant mutation, making the cross potentially useful in genetically mapping *Sleepless*. Male B6 mice presumed heterozygous for *Sleepless* based on phenotype were mated to female BALB mice from the Jackson Laboratory to create F_1_ animals. F_1_ male mice showing a high wake phenotype (presumably carrying the mutation) were then crossed with wild-type female B6 mice to create 269 [B6×(BALBxB6)F_1_]N_2_ (N_2_) male progeny. Thus, the N_2_ mice produced represented a genetically heterogeneous population with which we hoped to investigate: 1) mapping of the *Sleepless* mutation, which we will not elaborate on here, 2) a genome wide analysis for linkage between multiple sleep-wake traits and genomic regions and 3) the functional relationships among different sleep-wake traits.

## Results and Discussion

### Comparison of Sleep-wake Traits

Full EEG and EMG recordings over 48 hrs were collected from each N_2_ animal and sleep-wake parameters, defined by visually characterizing each 10 second epoch as wake, NREM or REM sleep, and performing EEG spectral analysis, allowed for the measurement of 72 parameters (See Supporting Information) defining sleep structure and continuity as well as EEG waveform activity. Before undertaking any of the analyses presented here, we selected from the 72 parameters we measure 20 traits that are most commonly used in the literature to describe sleep in rodent models. Applying factor analysis [10] to 1000 bootstrapped samples of the 20 sleep-wake traits over the 24-hr period allowed for an unbiased identification of structure within the multitude of variables (Table 1 and Supporting Information Table S2 for the bootstrapped 95% confidence intervals). These factors clustered into five trait dimensions that represent state amount, sleep fragmentation, REM sleep traits, latency to REM or NREM sleep and relative EEG spectral power. These five factors validate and confirm our *a priori* expectations that there are distinct and separable aspects of sleep. A similar approach has recently been used on human data to identify three principal components of human sleep that involved 1) sleep duration, 2) NREM intensity and 3) sleep continuity [11]. Means and standard deviations of the 20 sleep traits in the N2 population are presented in Supporting Information Table S1.

**Table 1:** Factor Analysis of Sleep-Wake Traits.

Trait	Factor 1	Factor 2	Factor 3	Factor 4	Factor 5
	Fragmentation	REM Sleep	State Amount	Power Bands	Latency
nb Wake	**−0.82**	−0.20	0.03	0.26	−0.04
db Wake	**0.77**	0.22	−0.31	−0.23	0.09
nb NREM	**−0.96**	−0.07	0.19	−0.02	−0.08
db NREM	**0.95**	0.13	0.22	0.02	0.09
db TS	**0.96**	0.08	0.19	0.03	0.10
# Arousals	**−0.69**	0.13	0.27	−0.30	−0.08
# Shifts	**−0.95**	−0.13	0.19	−0.02	−0.09
Onset REM	**0.85**	0.16	0.33	0.04	0.11
REM min	−0.17	**−0.91**	−0.02	0.08	0.02
% REM/TS	−0.14	**−0.83**	−0.43	0.10	0.05
nb REM	−0.21	**−0.89**	0.05	0.00	−0.21
Inter REM	0.03	**0.85**	−0.09	0.05	0.19
Wake min	0.06	0.04	**−0.98**	0.05	0.08
NREM min	−0.03	0.12	**0.98**	−0.06	−0.09
NREM rel Delta	0.03	−0.02	0.01	**0.81**	0.03
REM rel Theta I	0.14	0.10	0.02	**−0.78**	0.00
REM rel Theta II	−0.07	−0.06	0.07	**−0.83**	0.02
lat NREM	0.18	−0.07	−0.01	−0.14	**0.86**
lat REM	0.10	0.18	−0.02	−0.02	**0.87**
db REM	0.04	0.14	−0.11	0.15	0.39
**Proportion of Total Variance Explained**	**31.4%**	**16.5%**	**12.9%**	**11.2%**	**9.1%**
				**TOTAL**	**81.1%**

Factor loadings (Statistica, StatSoft, Inc.) of the 20 sleep-wake variables in the 24-hr period are shown with the factor-determining loading values bolded (generally greater than ±0.75). The 5 factors account for 81 percent of the total variance in the data. See Supporting Information for complete trait descriptions, and Supporting Information Table S2 for a 1000× bootstrap-obtained estimate of the 95% confidence intervals of the factor loading values.

### Quantitative Trait Loci Analysis

**Figure 1:**
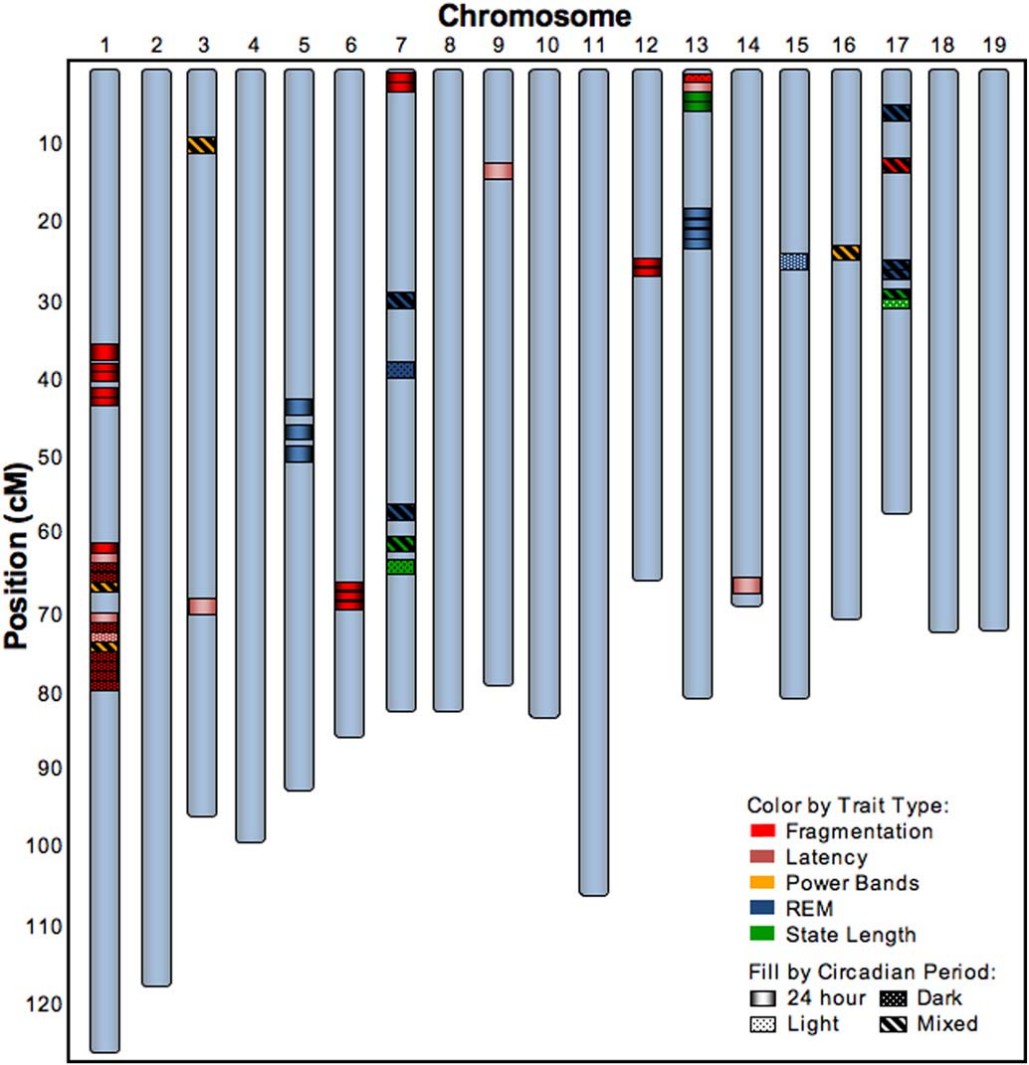
52 QTL for 20 sleep-wake traits. 52 QTL shown by chromosome and cM positions that were identified for each of the sleep-wake traits listed in Table 1. The colored bands represent the position of the peak LOD score for each QTL and the fill of the bands denote the time period for the trait linkage as shown in the insert legend. Based on the factor analysis depicted in Table 1, the traits are grouped into 1 of 5 categories designated by the color of the bands as noted in the insert legend. The precise peak (in cM and Mb) and LOD score of the QTL, as well as the specific sleep-wake trait represented by each of the colored bands, are provided in Table 2. Further information on the size of the QTL are provided in Supporting Information Table S3.

Linkage analysis was conducted with a set of 2,310 informative SNPs across the 19 autosomes from 269 N2 mice for which both complete and high quality genotype and sleep-wake phenotype data were obtained. The 48-hr sleep recording period was partitioned into two 24-hr periods and further into a light and dark phase yielding four recording time domains per animal during which each sleep trait was computed (see Supporting Information for further details on the statistical methods). Linkage analysis revealed a total of 52 significant QTL (comprising a minimum of 20 genomic loci) for the traits studied in this cross with LOD scores ranging from 2.5 to 7.6 (Fig. 1 and Table 2). Over half of these (28) reflected trait variation occurring across the full 24-hr day, indicating that much of the genetic control of sleep acts consistently across the light and dark periods. However, 12 additional QTL, termed “mixed-effect QTL”, reflected trait variation across the full 24-hr period where the direction and/or magnitude of the effect of the locus on the trait is statistically different between the light and dark periods. This indicates that the genotype at a locus can have the opposite or a quantifiably different effect on the same trait during the light versus the dark phase. In some cases, this effect was quite dramatic, as was observed for the wake min QTL at Q17@29 (LOD 7.6) where the estimated effect of the BALB genotype at this locus was 8.1 min in the light but −24.7 min in the dark (Supporting Information Table S3). Finally, some QTL were only detected in the dark (N = 9) or in the light (N = 3), indicating that the genotype at some loci only influenced sleep-wake traits during certain periods of the 24-hr day.

**Table 2:** Peak Location and LOD Score of Each QTL Depicted in Figure 1.

Name	Trait	Trait_Type	Chr	Peak (cM)	Peak (Mb)	LOD	FDR*	Period
Q1@39	Onset REM	Fragmentation	1	38.470	109.044	4.445	.006	24 hour
Q1@39	db TS	Fragmentation	1	39.120	115.677	3.453	.034	24 hour
Q1@39	db NREM	Fragmentation	1	39.120	115.677	3.438	.034	24 hour
Q1@39	nb NREM	Fragmentation	1	42.400	133.902	3.485	.026	24 hour
Q1@39	# Shifts	Fragmentation	1	42.400	133.902	3.076	.033	24 hour
Q1@64	# Arousals	Fragmentation	1	62.230	172.886	4.231	.004	24 hour
Q1@64	lat REM	Latency	1	62.230	172.886	2.941	.046	24 hour
Q1@64	db Wake	Fragmentation	1	62.230	172.886	3.991	.019	Dark
Q1@64	nb Wake	Fragmentation	1	63.680	176.451	3.285	.018	Dark
Q1@64	REM rel Theta I	Power Bands	1	63.680	176.451	2.790	.040	Mixed
Q1@75	lat NREM	Latency	1	74.200	187.588	4.528	.001	24 hour
Q1@75	db TS	Fragmentation	1	74.200	187.588	3.252	.023	Dark
Q1@75	lat NREM	Latency	1	74.200	187.588	2.796	.019	Dark
Q1@75	NREM rel Delta	Power Bands	1	74.200	187.588	3.097	.044	Mixed
Q1@75	Onset REM	Fragmentation	1	74.580	188.870	3.475	.009	Dark
Q1@75	db NREM	Fragmentation	1	76.960	189.312	3.176	.027	Dark
Q1@75	# Arousals	Fragmentation	1	76.960	189.312	2.493	.041	Dark
Q3@12	NREM rel Delta	Power Bands	3	12.180	30.690	2.745	.042	Mixed
Q3@71	lat NREM	Latency	3	71.060	146.067	3.244	.027	24 hour
Q5@49	% REM/TS	REM	5	45.290	65.322	4.225	.008	24 hour
Q5@49	db REM	REM	5	49.320	70.388	6.339	.001	24 hour
Q5@49	REM	REM	5	51.940	73.499	4.224	.005	24 hour
Q6@70	# Shifts	Fragmentation	6	70.000	135.767	3.111	.040	24 hour
Q6@70	nb NREM	Fragmentation	6	70.000	135.767	3.079	.033	24 hour
Q6@70	db Wake	Fragmentation	6	70.000	135.767	3.666	.024	24 hour
Q7@1	db Wake	Fragmentation	7	0.980	7.882	3.113	.045	24 hour
Q7@1	nb Wake	Fragmentation	7	0.980	7.882	4.064	.012	24 hour
Q7@32	nb REM	REM	7	31.770	50.217	4.288	.002	Mixed
Q7@40	REM min	REM	7	40.200	67.412	3.608	.004	Light
Q7@63	Inter REM	REM	7	59.900	102.969	4.014	.046	Mixed
Q7@63	Wake min	State Length	7	62.850	114.066	4.812	.022	Mixed
Q7@63	NREM min	State Length	7	65.140	114.924	4.443	.027	Light
Q9@15	lat NREM	Latency	9	15.160	46.527	3.062	.026	24 hour
Q12@28	db TS	Fragmentation	12	27.580	71.050	3.111	.035	24 hour
Q12@28	db NREM	Fragmentation	12	27.580	71.050	3.119	.036	24 hour
Q13@2	# Arousals	Fragmentation	13	0.000	4.692	2.536	.049	Light
Q13@2	lat REM	Latency	13	1.640	14.548	2.829	.042	24 hour
Q13@2	Wake min	State Length	13	1.970	16.724	5.139	.001	24 hour
Q13@2	NREM min	State Length	13	1.970	16.724	3.593	.049	24 hour
Q13@23	db REM	REM	13	20.030	60.142	3.299	.026	24 hour
Q13@23	REM min	REM	13	22.660	68.690	6.302	<.001	24 hour
Q13@23	% REM/TS	REM	13	22.660	68.690	6.473	<.001	24 hour
Q13@23	Inter REM	REM	13	25.940	72.819	3.928	.012	24 hour
Q14@73	lat REM	Latency	14	72.980	121.782	3.058	.046	24 hour
Q15@26	% REM/TS	REM	15	26.300	82.415	4.040	.002	Dark
Q16@24	REM rel Theta I	Power Bands	16	23.660	51.539	3.039	.037	Mixed
Q17@13	% REM/TS	REM	17	3.940	24.849	4.263	.002	Mixed
Q17@13	db TS	Fragmentation	17	13.120	44.789	2.601	.049	Mixed
Q17@29	nb REM	REM	17	28.590	65.200	3.021	.014	Mixed
Q17@29	REM min	REM	17	28.590	65.200	4.870	<.001	Mixed
Q17@29	Wake min	State Length	17	30.230	65.622	7.562	<.001	Mixed
Q17@29	NREM min	State Length	17	30.230	65.622	5.954	.001	Dark

QTL were detected by R package QTL as were the peak LOD scores. The QTL peak was defined as the position within the QTL with the highest LOD score. Trait type was determined by factor analysis (Table 1). Chr = chromosome, LOD = logarithm of odds. FDR = False Discovery Rate (probability). See Supplementary Information for complete trait descriptions. See Supporting Information Table S3 for further information about each QTL.

* To account for multiple testing using a non-parametric approach, FDR estimates were computed genome-wide within each sleep trait by permuting individual identifiers for the genotype data and repeating the analyses on 1000 replicate permuted data sets [25]. To eliminate the influence of markers in high linkage disequilibrium, chromosome-wide peak marker LOD scores were used for all FDR computations.

We found that the QTL associated with any specific trait can be highly time- dependent, adding another dimension to the richness of the genetic landscape underlying sleep-wake traits. For example, while REM min over 24 hrs mapped to Q5@49 (LOD 4.2) and Q13@23 (LOD 6.3), REM min during the light mapped to Q7@40 (LOD 3.6), while a mixed effect on REM min mapped to Q17@29 (LOD 4.9). We also found that different trait groups show a bias as to whether they are affected by QTL in a similar manner across the 24-hr day, as opposed to QTL having different effects on the traits in the light versus the dark. For example, 19 of the 21 fragmentation QTL (Fig. 1, red symbols) were linked to the trait in the dark period or over the full 24 hrs, while all 4 of the EEG power band QTL fell in the mixed-effect category (Fig. 1, yellow symbols). These findings indicated that the genetic regulation of a single sleep-wake trait was highly dependent on circadian time or the environmental light-dark cycle.

Analysis of such a large genotype/phenotype data set allowed us to observe intricacies in the genetic landscape in the control of specific sleep-wake traits not previously detected. For example, while both db REM and nb REM together determined the total amount of REM sleep, these two REM traits were at least partially under differential genetic control (Fig. 1), since QTL for db REM mapped to Q5@49 (LOD 6.3) and Q13@23 (LOD 3.3), while QTL for nb REM mapped to Q7@32 (LOD 4.3) and Q17@29 (LOD 3.0). While it may be expected that QTL for mathematically related sleep-wake traits (e.g. measures of fragmentation such as nb NREM and db Wake) might map to the same region, QTL for less directly related traits (e.g. # Arousals and NREM delta power, or wake min and REM latency) might not have been expected to map to the same loci as occurred in Q1@75 (LOD 2.5–4.5) and Q13@2 (LOD 2.5–5.1) (Table 2). These unexpected genetic relationships raise the possibility that shared genetic regulatory mechanisms may underlie different sleep-wake traits that were not previously thought to be related.

It should be noted that two genomic regions, on chromosomes 7 and 13, were associated with high wake and the B6 genotype. As the *Sleepless* mutation has a high wake phenotype, these represent candidate regions for the mutation. In addition, the presence of the *Sleepless* mutation segregating in this N_2_ population may have influenced the genetic effect of some of our identified QTL. Previous studies have shown that the presence of a mutation can reveal epistatic effects of other genes that may otherwise not be apparent [12]. Therefore some of the specific QTL presented here might be present because the locus represents *Sleepless* or because it is a region that interacts with *Sleepless*. However, analysis of epistatic interactions with these two regions failed to identify any significant interactions with the region on chromosome 13, and only one region of significant interaction with chromosome 7 (see Supporting Information Figure S2). Thus, it appears the presence of *Sleepless* segregating in the N2 population influenced the QTL results at most at two loci, if the *Sleepless* locus is not normally polymorphic between B6 and BALB.

### Bayesian network analysis

**Figure 2:**
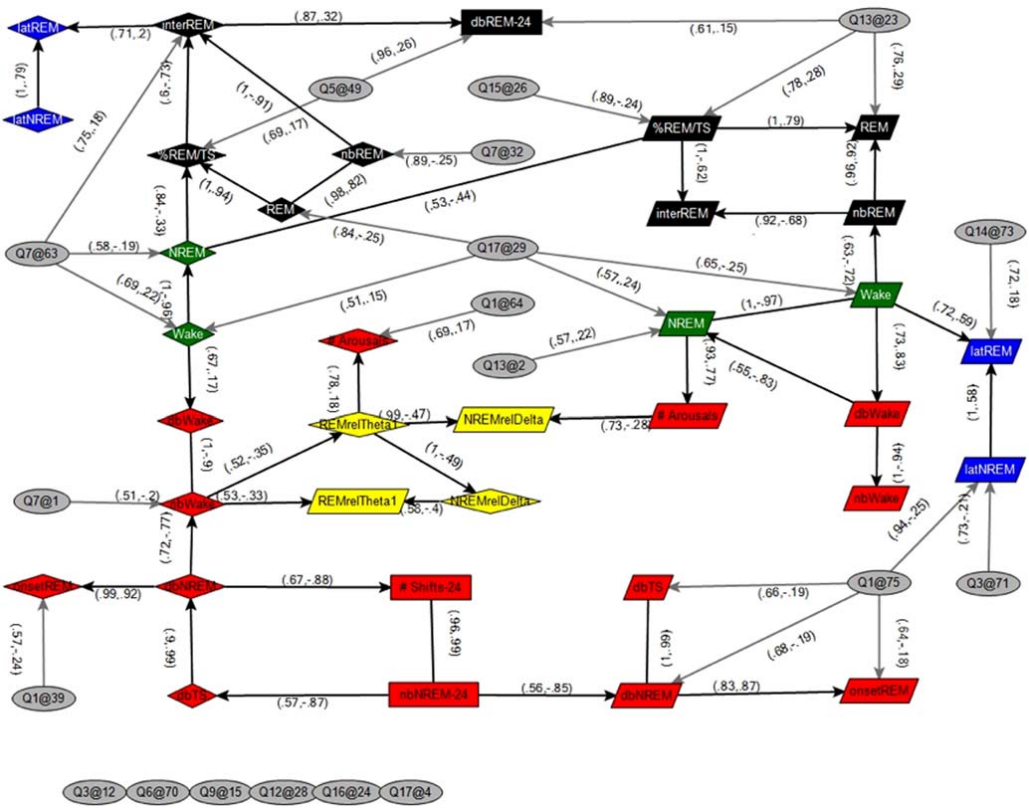
QTL and Sleep-wake Trait Network Analysis. Bayesian network of sleep traits and significant QTL. Sleep-wake traits with QTL that differed in effects between the light and dark periods were represented in the network as two distinct traits, measured in the light and dark periods, denoted by diamond shapes (and suffix ‘l’) and parallelograms (and suffix ‘d’), respectively. The remaining sleep traits were averaged over the light and dark periods, denoted by rectangles (and suffix ‘24’). QTL are represented by ellipses, <chromosome>@<centiMorgan>. Edge labels include the confidence, which is the proportion of 1500 Markov Chain Monte Carlo (MCMC) learned networks that included the edge, and the Pearson correlation between nodes, respectively. Node colors represent trait types described in text; green: wake, black: REM, red: fragmentation, yellow: power band and blue: latency to REM. Edges that were present in greater than 50 percent of the MCMC runs were included in the network. Details of Bayesian network construction can be found in Supporting Information.

In order to create a dynamic model that allows for the visualization of relationships among multiple sleep phenotypes and multiple sleep QTL in a context specific manner we used Bayesian analysis. Construction of a Bayesian network facilitated visualization of the strongest links between the sleep phenotypes and QTL and identification of sub-networks and patterns defined by those links that otherwise might not be seen [13], [14]. While some links are obvious (e.g. a QTL causing decreased wake over 24 hrs can be expected to cause an increased amount of NREM over 24 hrs), the network revealed a number of less intuitive links. Using a stringent set of criteria by limiting the identification of nodes and edges (Supporting Information), Fig. 2 exposes a network of the strongest statistically defined interactions between different sleep-wake traits during the light, dark or full 24-hr period, as well as between these traits and the QTL identified in this cross. Trait by trait correlations (Supporting Information Table S4) and a less stringent Bayesian network (Supporting Information Fig. S1) are also provided. Incorporating this large amount of data into one cohesive network represents a novel model for understanding the complex behavior of sleep and the interactions of sleep-wake traits at the genetic and trait by trait levels.

One of the most salient features of the network shown in Fig. 2 was the clustering of sleep phenotypes into sub-networks according to trait type. The sub-networks are comprised of small, interconnected groups of traits that, in this case, align with the previously defined loading factors (Table 1). The most conserved sub-network by trait type in both the light and dark phases was REM sleep. Between both phases, the pattern was maintained with arcs leading from % REM/TS and nb REM to inter-REM interval. In fact, all trait nodes were directly linked to other nodes of the same trait type, with only one exception. The nodes for number of brief arousals, in the light and dark phases, were detached from other fragmentation nodes, although in both cases there was only a single degree of separation. The connection of brief arousals to power bands may be a reflection of the links between sleep depth and excitability [5]. The link between sleep continuity and intensity in our network is particularly interesting in view of the finding that in two different gene deletion models (prion protein and alpha1G T-type Ca2+ channels) there is a decrease in NREM power density accompanied by an increase in the number of brief awakenings [15], [16].

Aside from power band and fragmentation factors, there was only one edge linking a light and dark node in Fig. 2, NREM min in the light with % REM/TS in the dark. This one clear edge led us to take a closer look at wake time in the light (or dark) phase with REM sleep time in the dark (or light phase). Such an analysis led to the surprising discovery that while there was no significant correlation between REM time within the light versus the dark phases, there were significant correlations such that the amount of wake in the dark was connected with the amount of REM sleep in the light, while the amount of wake in the light was correlated with the amount of REM in the dark (Supporting Information Table S4). Such an unexpected relationship raises a number of new questions about the relationship of wake time to subsequent REM sleep and/or how REM sleep time effects subsequent wake time. Such relationships are particularly intriguing in view of the possible role of REM sleep on a variety of higher brain functions, including learning [17], [18], memory [19], and mood [20], [21], as well as the possible genetics underlying these relationships.

## Materials and Methods

### Animals and Housing

All experimental mice were housed and handled according to the Federal Animal Welfare guidelines and all studies were approved in advance by the Animal Care and Use Committee at Northwestern University. All animals were maintained continuously on a 14 hr light: 10 hr dark cycle (LD 14∶10) from birth at a room temperature of 23±2°C and were weaned at 4 weeks of age. Food and water were available *ad libitum*.

All animals used for genotype and phenotype (N = 269) analysis were N2 offspring from 26 male F1 mice that were themselves the progeny of female BALB/cByJ (BALB) from the Jackson Laboratory and 11 C57BL/6J (B6) male mice (G4–6 males) from a line of B6 mice maintained at Northwestern University. This line descended from a single first generation B6 male offspring of a male mouse treated with the chemical mutagen *N*-ethyl-*N*-nitrosourea. This line was maintained as a possible mutant line with an altered sleep-wake phenotype because it showed increased wake (820±21 min) over 24 hours compared to wild-type B6 males (718±49 min), and hence the line was called *Sleepless*. The high wake phenotype in this line segregates as a single locus autosomal dominant mutation, and has been maintained by backcrossing affected males to wild-type B6 females obtained from the Jackson Laboratory at each generation to eliminate other possible induced mutations segregating in the line. The 11 B6 males used in the first generation cross for this study were high wake males from the 4^th^–6^th^ generation of *Sleepless* animals (and thus presumably, *Sleepless* heterozygotes) which were then crossed to BALB females from Jackson Laboratories to produce 124 (BALB×B6*)F1 males. F1 males were screened and examined for sleep-wake phenotype, and 26 animals selected for high wake minutes as presumptive *Sleepless* heterozygotes were backcrossed to wild-type B6 females from Jackson Laboratories to produce 269 [B6×(BALB×B6*)F1]N2 males. Thus, in addition to a 50∶50 chance of carrying the induced *Sleepless* mutation, the 269 N2 mice each have a 50∶50 probability of being either homozygous B6/B6 or heterozygous BALB/B6 at any genomic region. Therefore, in this cross of two inbred mouse strains, genetic polymorphisms can be used to map segregation of sleep-wake traits.

### Sleep-wake recordings in adult mice

At 10 to 12 weeks of age, male mice were prepared for monitoring of EEG/EMG signals [22]. A minimum 10-day post-surgery recovery period was observed before sleep recording was initiated. Mice were acclimated to housing individually in cylindrical (25.5 cm diameter) sleep recording cages with free access to food and water for a minimum of five days during this time. EEG/EMG data were collected [22] for 48 continuous hours starting at light onset. With the use of a custom software package (SleepReport, Actimetrics, Evanston, IL), EEG and EMG recordings were divided into 10-second epochs and scored via visual inspection as either wake, NREM or REM. For a detailed account of sleep and EEG analysis see Supplemental Information.

### Genotyping

All DNA samples were genotyped on the Affymetrix MegAllele™ genotyping mouse 5 K SNP panel: (http://www.affymetrix.com/support/technical/datasheets/parallele_mouse5k_datasheet.pdf). This panel consists of approximately 5,500 SNPs evenly distributed across the genome with approximately 2,310 of these SNPs being informative for the B6 and BALB inbred lines. DNA was prepared from mouse tail using the DNAeasy kit according to the manufacturer's protocols (Qiagen). Tails were stored frozen until DNA preparation, and DNA was stored at −20°C. DNA was quantified for quality control by fluorometry using PicoGreen (Invitrogen). It was shipped on dry ice and concentration adjusted per the manufacturer's instructions prior to genotyping. Expected genotype probabilities were computed using the R package QTL R/qtl: QTL with Haldane's map function [23]. Details on computing the expected genotype probabilities can be found in Supporting Information.

### Network Analysis

A Bayesian network is a directed acyclic graph (DAG) which includes a collection of nodes and arcs connecting nodes [24]. The nodes represent random variables and the arcs represent conditional probabilistic dependency between nodes, where the distribution of each node is dependent on its parent nodes but conditionally independent of all other nodes. Thus, Bayesian networks are constructed to represent not only correlation but causality. That is, X is a parent of Y, or the presence of a directed path from node X to node Y implies X causes (controls) Y. Thus, the network structure allows one to distinguish between the simple correlation or clustering and the more interesting notion of directed or causal dependence. Details of the construction of the network presented here are found in the Supplemental Information.

## Supporting Information

Text S1 (0.05 MB DOC)

Figure S1 Expanded Bayesian Network
(0.23 MB DOC)

Figure S2 Epistasis LOD Plot
(0.04 MB DOC)

Table S1 Sleep-Wake Traits in Light and Dark Periods
(0.06 MB DOC)

Table S2 Bootstrap Obtained 95% Confidence Intervals of Factor Analysis
(0.09 MB DOC)

Table S3 Expanded QTL Data
(0.31 MB DOC)

Table S4 Correlation Tables
(0.07 MB DOC)

Table S5 Bootstrap Results for Bayesian Network
(0.03 MB DOC)
