# Supplementary material for: Uncovering the Genetic Landscape for Multiple Sleep-Wake Traits
Source: PLoS One. 2009 Apr 10;4(4):e5161. doi: 10.1371/journal.pone.0005161 (PMC2664962; doi:10.1371/journal.pone.0005161)
Supplement: Text S1 — (0.05 MB DOC) [file pone.0005161.s001.doc]

**Supporting Information Text: Data Analysis**

to Accompany Winrow et al., 08-PONE-RA-06401R1

- 1. Sleep and EEG Analysis 2
  2. Definition of Sleep-Wake Measurements 3-5
  3. Expected Genotype Probabilities 6-7
  4. Construction of Bayesian Network 8-10

**1. Sleep and EEG analysis:**

Wake (low voltage, high frequency EEG and high amplitude EMG), non rapid eye movement sleep (NREM) (high voltage, low frequency EEG and low amplitude EMG) or rapid eye movement sleep (REM) (low amplitude EEG constituted mainly by theta wave activity and EMG atonia). Post-scoring analysis included the determination of sleep structure parameters, including sleep amount, the distribution and consolidation of sleep, and EEG spectral analysis. Sleep-wake parameters were calculated for all parameters for the individual 14-hr light and 10-hr dark periods, as well as for the total 24-hr day across a 48-hr period. Sleep amounts were determined by summing all epochs of a state in the given period. Sleep architecture was assessed by determining the number of arousals from sleep (a 10-second epoch of wakefulness bounded by NREM or REM sleep), stage shifts (number of transitions between ten second epochs of wake, NREM, and REM), sleep/wake bouts (at least 2 consecutive epochs, 20 seconds, of wake NREM, REM or total sleep) and the average duration (minutes) of sleep/wake bouts.

For quantitative analysis of the EEG signal, each 10-second scoring epoch was divided into five 2-second intervals and subjected to Fast Fourier Transformation, which included a range of 1-30 Hz. For all epochs of wake, NREM and REM sleep, the EEG power in the delta (1-4 Hz), theta I (4-8 Hz), theta II (8-11 Hz), sigma (11-15 Hz) and beta (15-30 Hz) frequency ranges were calculated. This processes yielded values of both absolute and relative power in each of the frequency bands.

**2. Definition of Sleep-Wake Measurements**

**72 sleep-wake parameters**

(*) Indicate traits included in presented analysis

1. State Amount

An epoch is defined as 10 seconds

## *Wake min sum of all Wake epochs in minutes

*NREM min sum of all non-rapid eye movement (NREM) epochs in minutes

TS min Total sleep (NREM + REM) time in minutes

W % Percentage of recording time in Wake

NREM % Percentage of recording time in NREM

TS % Percentage of recording time in TS

2. REM Sleep

*REM min sum of all rapid eye movement (REM) epochs in minutes

REM % Percentage of recording time in REM

% NREM/TS Percentage of TS time in NREM

*% REM/TS Percentage of TS time in REM

*nb REM Number of REM bouts

*db REM Mean duration (or length) of REM bouts in minutes

*Inter REM Inter REM interval – average number of minutes between REM bouts (from the end of one REM bout to the beginning of the next REM bout)

3. Fragmentation

A state episode (or bout) requires at least 2 consecutive epochs (20-sec duration)

*nb Wake Number of NREM bouts

*db Wake Mean duration (or length) of Wake bouts in minutes

*nb NREM Number of NREM bouts

*db NREM Mean duration (or length) of NREM bouts in minutes

nb TS Number of TS bouts

*db TS Mean duration (or length) of TS bouts in minutes

*# Arousals Number of brief arousals of wake (single epochs) bounded by sleep

*# Shifts Total number of state shifts (or transitions)

*onset REM Average number of minutes from the start of a sleep bout (beginning with NREM sleep) to the start of a REM bout within that sleep bout

4. Latency

*lat NREM Number of minutes from the beginning of a time domain (light or dark onset) to the beginning of the first bout of NREM sleep

*lat REM Number of minutes from the beginning of a time domain (light or dark onset) to the beginning of the first bout of REM sleep

5. Power Bands (Sleep Intensity)

Sleep intensity variables are measured with state specific EEG power spectral analysis (FFT: Fast Fourier Transform) in W, NREM and REM.

Each column is labeled with the format of “state, type of power, band.” For example “NREM rel Theta II” would be the relative power of theta II during NREM sleep. A description of each of these parameters follows:

**Absolute power (abs P)** density values are the average power density (calculated per 2-sec sub- epoch) in each frequency band.

**Relative power (rel P)**: Once the absolute power is calculated, it can then be determined what percentage (%) of overall power (the entire frequency range) is comprised of the individual frequency bands. Because relative power is calculated as a percentage, the sum of relative power across the 5 frequency bins should equal 100%. It provides information on the distribution of the frequency bands.

**Total energy (EN):** power density values in each frequency band summed over all epochs in the entire recording period, and characterize sleep/wake amount (time and intensity).

**Power spectra values are divided into 5 frequency bands for each measurement:**

***B1: Delta (1.0 - 4.0 Hz)** Delta waves predominate in NREM sleep and are associated with EEG sleep intensity or sleep “depth”. Delta activity is normally elevated during NREM sleep that follows prolonged periods of wakefulness (i.e. either after normal waking or after experimental sleep deprivation). The delta frequency range in normally set at 0.5 – 4 Hz or 1 – 4 Hz in the majority of human and animal studies. (NREM rel Delta power is included in the analysis)

***B2: Theta I (4.0 – 8.0 Hz)** see B3: Theta II

***B3: Theta II (8.0 – 11.0 Hz)** Theta activity is the major EEG frequency generated during REM sleep. In humans, theta activity is consistently set at 4 – 7 Hz. However, in animal studies, there is less consistency and a larger range is commonly used. Therefore, we have included 2 frequency bands for theta activity, on in the lower frequency range (4- 8 Hz) and one in the higher frequency range (8 – 11 Hz). (REM rel Theta I & II power are included in the analysis)

**B4: Sigma (11.0 – 15.0 Hz)** The sigma (11 – 15 Hz) frequency band indicates EEG spindling activity, which is indicative of NREM sleep in humans and rodents. In humans, “sleep spindles” are distinct EEG events (bursts of 12-14 Hz activity lasting .5 – 2 seconds in duration) that occur during NREM (Stage 2) sleep. The occurrence of sleep spindles are often used to signify the transition from wakefulness to sleep. In rodents, the sigma activity is robust during NREM sleep, however, does appear as distinct EEG events. Sigma waves appear more intermixed with delta and theta waveforms. A trademark of sigma activity is that it is increased by benzodiazepine administration.

**B5: Beta (15.0 – 30.0 Hz)** Beta activity represents the upper limit of our EEG frequency range. Again, differences in the definition of this band can be used, and we have selected 15 – 30 Hz.

There are a total of 72 parameters available for data analysis. These parameters are available across the entire two-day time frame (baseline one (BL1) and two (BL2)) in two hour intervals. We also summate the data into 14-hr light phase, 10-hr dark phase, and 24-hr total for BL1 and BL2.

**3. Expected genotype probabilities**

Expected genotype probabilities were computed using the R package QTL R/qtl: QTL with Haldane's map function [1]. With such a high density panel of SNPs, it was not necessary to evaluate the model between markers, but the Haley-Knott approach allowed us to resolve no-calls and double-crossovers, which were set to missing. The mixed models were fitted using the R package NLME [2]. Fixed effects were included in the model to adjust for the possible effects of mouse age at sleep trait recording, father, sleep scorer, and calendar month of recording. Outliers more than 3 interquartile ranges from the first or third quartiles were removed.

For the model, the sleep trait measurement, *Yij*, for the *i*th individual in the *j*th time domain was regressed on indicators for light phase, *l*, marker genotype, *L*, light phase-marker interaction, and a vector of fixed covariates, *X*, in the model,

where correlations between observations within an individual are estimated with the six ρ parameters. The model was fitted with and without the main-effect marker term and the interaction term. Likelihood ratio tests were conducted to identify statistically significant QTL and to determine whether the QTL effects differed significantly between light and dark phases. Both start and end QTL boundaries were defined by two adjacent markers both with LOD scores of less then one. To control the trait-specific False Discovery Rate (FDR) at the 0.05 level, permutation tests were conducted by randomizing individual animal labels with respect to genotypes for each of 1000 genome-wide linkage analyses, where the QTL peak score was defined as the chromosome maximum.

1. Broman, KW, Wu H, Sen S, & Churchill GA. (2003) R/qtl: QTL mapping in experimental crosses. *Bioinformatics* **19**, 889-90
2. Pinheiro J and Bates DM. (2000) Mixed-Effects Models in S and S-Plus. New York: Springer.

**4. Construction of the Bayesian Network**

We used a Markov Chain Monte Carlo (MCMC) algorithm called Metropolis-Hastings (MH) to globally search the space of all DAGs (Directed Acyclic Graphs). In this procedure, the network was initially devoid of edges and then edges were added, removed, or reversed in direction randomly, then the network score was computed and compared with the previous state of the score. The simulated annealing algorithm was used to determine if this action was acceptable or not. This process was repeated until the score or the network structure became stable. This procedure collected results for a set of networks, a Markov Chain, which was one MCMC run. The network with the highest score from a MCMC run was considered a potential DAG best fit to the data locally. However, due to the huge number of possible DAGs when n is large, a MCMC run may still be fixed to a local score optimum, thus, it was necessary to carry out multiple MCMC runs and to select edges repeatedly enriched in the learned networks. We used 0.5 as a threshold of enrichment of edges to determine whether the edge was acceptable or not for the final network, e.g., for 1000 MCMC runs, if arc i -> j or j -> i appears more than 500 times, we accepted the edge between i and j. To determine edge direction, we also selected by enrichment: the edge direction was i -> j if number of (i -> j) arcs was greater than the number of (j -> i) arcs by 3 standard deviations or 10 %. Direction was undetermined if there was no enrichment of one direction over the other.

In building our network, we used the framework suggested by Bøttcher and Dethlefsen [3] to treat the local and joint probability distributions and compute network scores. The DEAL [3] software package for analyzing data using Bayesian networks handles both discrete and/or continuous nodes when the number of nodes is small. We implemented a MCMC search strategy using the DEAL scoring methods. Our software handled up to 10,000 nodes (continuous and/or discrete). We compared our result with that by DEAL on small networks, and found they were compatible. We also carried out extensive simulations for large networks (approximately 5,000 nodes). In the case where all nodes were either discrete or discretized continuous nodes, our software was able to give a satisfactory reconstruction of large networks [4]. Here we applied the method more generally to reconstruct a network that included both discrete and continuous nodes. Our simulations also showed that we could obtain a satisfactory recovery rate for this case as well.

For the present study, we used our software to construct a Bayesian network of 19 QTL (discrete nodes) and 35 traits (continuous nodes) controlled by those QTL. QTL were explicitly represented in the network by QTL peak markers such that any 10 cM region of chromosome was represented by at most one genetic marker. Traits that were found to be differentially genetically controlled between the light and dark phases were partitioned into two traits for network reconstruction, a light phase trait, averaged over the two 24 hr cycles, and a dark phase trait. Traits that did not differ significantly in QTL effects across the light and dark phases were included as a single 24 hr trait (except latency to REM, which was partitioned into light and dark phase traits due to the distinct nature of the phenotype with respect to phase). Trait nodes were constrained to have at most two trait parent nodes in order to pick up the most significant interactions. There were no limitations on the number of QTL parent nodes. Edges were banned between QTL and traits that were not found to be controlled by those respective QTL in marker-by-marker linkage analysis (Table 2). Edges were banned between phase partitions of the same trait, e.g., an edge was banned between light phase wake and dark phase wake. The final network was reconstructed based on 12,000 independent MCMC runs.

We further explored the robustness of the network by employing a bootstrap approach to estimate additional summary statistics for each observed edge (Table S5). One thousand replicate bootstrap datasets were generated yielding 1000 estimated Bayesian networks, each based on 1500 MCMC runs. In most cases, the median bootstrap consistency agreed with the observed consistency cutoff of .5. Also, bootstrap results for edge direction agreed well with the observed network. As a whole, the bootstrap results support the structure of the observed network.

1. Bottcher SG & Dethlefsen C (2003) deal: A package for learning Bayesian networks. Journal of Statistical Software 8: 1-40.
2. Zhu J, Weiner MC, Zhang C, Frigman A, Minch E, et al. (2007) Increasing the power to detect causal associations by combining genotypic and expression data in segregating populations. PLoS Comput Biol 3:e69.
