# Supplementary material for: Uncovering the Genetic Landscape for Multiple Sleep-Wake Traits
Source: PLoS One. 2009 Apr 10;4(4):e5161. doi: 10.1371/journal.pone.0005161 (PMC2664962; doi:10.1371/journal.pone.0005161)
Supplement: Figure S1 — Expanded Bayesian Network (0.23 MB DOC) [file pone.0005161.s002.doc]

## **Supporting Information**

To accompany Winrow et al., 08-PONE-RA-06401R1

## **Uncovering the Genetic Landscape for Multiple Sleep-Wake Traits**

**Figure S1: Expanded Bayesian Network**

Bayesian network of sleep traits and significant QTL. Constraints were relaxed from the original network (Fig. 2) to allow up to 10 parents per node (instead of 2). Sleep traits with QTL that differed in effects between the light and dark periods were represented in the network as two distinct traits, measured in the light and dark periods, denoted by diamond shapes (and suffix 'l') and parallelograms (and suffix 'd'), respectively. The remaining sleep traits were averaged over the light and dark periods, denoted by rectangles (and suffix '24'). QTL are represented by ellipses, <chromosome>@<centiMorgan>. Node colors represent trait types described in text; green:wake, black:rem, red:fragmentation, yellow:powerband and blue:latencyREM. Edges that were present in greater than 50 percent of the MCMC runs were included in the network..
