# Supplementary material for: Uncovering the Genetic Landscape for Multiple Sleep-Wake Traits
Source: PLoS One. 2009 Apr 10;4(4):e5161. doi: 10.1371/journal.pone.0005161 (PMC2664962; doi:10.1371/journal.pone.0005161)
Supplement: Figure S2 — Epistasis LOD Plot (0.04 MB DOC) [file pone.0005161.s003.doc]

## **Supporting Information**

To accompany Winrow et al., 08-PONE-RA-06401R1

## **Uncovering the Genetic Landscape for Multiple Sleep-Wake Traits**

**Figure S2: Epistasis LOD Plot.** The LOD (logarithm of the odds ratio) scores for interaction between genetic loci on chromosome 7 with other loci genome-wide (red) and between genetic loci on chromosome 13 and other loci genome-wide (black) are shown. A significant interaction between chromosome 4 and chromosome 7 was detected with a LOD score of 4.26. No other interactions with either the chromosome 7 or the chromosome 13 regions were detected with LOD scores above 2. Epistasis analysis was done using the “scantwo” function in R/qtl software.
