# Supplementary material for: Uncovering the Genetic Landscape for Multiple Sleep-Wake Traits
Source: PLoS One. 2009 Apr 10;4(4):e5161. doi: 10.1371/journal.pone.0005161 (PMC2664962; doi:10.1371/journal.pone.0005161)
Supplement: Table S1 — Sleep-Wake Traits in Light and Dark Periods (0.06 MB DOC) [file pone.0005161.s004.doc]

## **Supporting Information**

To accompany Winrow et al., 08-PONE-RA-06401R1

## **Uncovering the Genetic Landscape for Multiple Sleep-Wake Traits**

**Table S1: Sleep-Wake traits in Light and Dark Periods**

Mean and standard deviations for the entire N2 population for each of the 20 sleep-wake traits during the entire 24-hr period, the Light period, and the Dark Period are shown.

|  | **24 hour** | | **14 hr Light** | | **10 hr Dark** | |
| --- | --- | --- | --- | --- | --- | --- |
| **Trait** | **Mean** | **Std.Dev** | **Mean** | **Std Dev** | **Mean** | **Std Dev** |
| **Wake min** | 708.5 | 60.9 | 314.5 | 37.4 | 394.2 | 58.2 |
| **NREM min** | 666.0 | 60.7 | 477.1 | 36.2 | 188.9 | 52.1 |
| **REM min** | 61.5 | 11.2 | 48.4 | 9.8 | 13.1 | 5.6 |
| **% REM/TS** | 8.5 | 1.7 | 9.2 | 1.8 | 6.37 | 1.99 |
| **nb Wake** | 146.5 | 42.1 | 102.5 | 29.6 | 44.7 | 18.5 |
| **db Wake** | 5.18 | 1.81 | 3.19 | 1.03 | 11.50 | 7.67 |
| **nb NREM** | 261.2 | 59.5 | 185.7 | 37.9 | 75.6 | 30.1 |
| **db NREM** | 2.68 | 0.64 | 2.69 | 0.67 | 2.72 | 0.71 |
| **nb REM** | 56.9 | 10.5 | 44.1 | 8.9 | 12.8 | 5.1 |
| **db REM** | 1.07 | 0.13 | 1.09 | 0.15 | 1.39 | 4.32 |
| **Db TS** | 17.62 | 4.19 | 17.9 | 4.4 | 17.5 | 4.6 |
| **# Arousals** | 128.4 | 40.6 | 92.4 | 27.4 | 36.0 | 17.4 |
| **# Shifts** | 613.2 | 135.1 | 438.5 | 86.9 | 174.7 | 69.1 |
| **lat NREM** | 14.1 | 16.7 | 14.1 | 16.7 | 63.9 | 73.3 |
| **lat REM** | 34.8 | 24.7 | 34.8 | 24.7 | 132.9 | 90.1 |
| **Onset REM** | 3.38 | 0.85 | 3.31 | 0.87 | 4.00 | 4.38 |
| **Inter REM** | 23.8 | 6.7 | 17.8 | 10.7 | 39.8 | 24.7 |
| **NREM rel Delta** | 0.378 | 0.042 | 0.369 | 0.041 | 0.402 | 0.047 |
| **REM rel Theta I** | 0.396 | 0.048 | 0.397 | 0.048 | 0.390 | 0.047 |
| **REM rel Theta II** | 0.203 | 0.045 | 0.201 | 0.044 | 0.209 | 0.051 |
