# Supplementary material for: Uncovering the Genetic Landscape for Multiple Sleep-Wake Traits
Source: PLoS One. 2009 Apr 10;4(4):e5161. doi: 10.1371/journal.pone.0005161 (PMC2664962; doi:10.1371/journal.pone.0005161)
Supplement: Table S2 — Bootstrap Obtained 95% Confidence Intervals of Factor Analysis (0.09 MB DOC) [file pone.0005161.s005.doc]

## **Supporting Information**

To accompany Winrow et al., 08-PONE-RA-06401R1

## **Uncovering the Genetic Landscape for Multiple Sleep-Wake Traits**

**Table S2: Bootstrap obtained 95% Confidence Intervals of Factor Analysis.**

The factor analysis was iteratively performed on 1000 bootstrapped-with-replacement samples of the original sleep-wake trait data. The upper 95% and lower 5% factor loading values from this analysis are shown with the same cells bolded as in Table 1.

Table S2: Bootstrap obtained 95% Confidence Intervals of Factor Analysis

| **Upper 95% Confidence Values** | Factor | Factor | Factor | Factor | Factor | **Lower 5% Confidence Values** | Factor | Factor | Factor | Factor | Factor |
| --- | --- | --- | --- | --- | --- | --- | --- | --- | --- | --- | --- |
|  | 1 | 2 | 3 | 4 | 5 |  | 1 | 2 | 3 | 4 | 5 |
|  | **Fragment ation** | **REM Sleep** | **State Amount** | **Power Bands** | **Latency** |  | **Fragment ation** | **REM Sleep** | **State Amount** | **Power Bands** | **Latency** |
| nb Wake | **-0.78** | -0.12 | 0.12 | 0.33 | -0.01 | nb Wake | **-0.84** | -0.28 | 0.00 | 0.18 | -0.14 |
| db Wake | **0.81** | 0.30 | -0.23 | -0.13 | 0.20 | db Wake | **0.72** | 0.16 | -0.39 | -0.31 | 0.01 |
| nb NREM | **-0.94** | -0.02 | 0.25 | 0.00 | -0.03 | nb NREM | **-0.97** | -0.12 | 0.13 | -0.08 | -0.11 |
| db NREM | **0.96** | 0.20 | 0.26 | 0.08 | 0.15 | db NREM | **0.93** | 0.09 | 0.15 | 0.00 | 0.02 |
| db TS | **0.97** | 0.14 | 0.23 | 0.08 | 0.15 | db TS | **0.94** | 0.03 | 0.12 | 0.00 | 0.02 |
| # Arousals | **-0.63** | 0.22 | 0.39 | -0.20 | -0.01 | # Arousals | **-0.74** | 0.03 | 0.17 | -0.40 | -0.18 |
| # Shifts | **-0.93** | -0.08 | 0.25 | 0.00 | -0.03 | # Shifts | **-0.96** | -0.19 | 0.13 | -0.08 | -0.12 |
| Onset REM | **0.88** | 0.23 | 0.39 | 0.11 | 0.17 | Onset REM | **0.81** | 0.10 | 0.25 | 0.00 | 0.01 |
| REM min | -0.09 | **-0.84** | -0.01 | 0.16 | 0.26 | REM min | -0.23 | **-0.95** | -0.15 | 0.01 | 0.01 |
| % REM/TS | -0.07 | **-0.76** | -0.32 | 0.17 | 0.20 | % REM/TS | -0.20 | **-0.89** | -0.54 | 0.01 | 0.01 |
| nb REM | -0.16 | **-0.84** | 0.15 | 0.10 | -0.02 | nb REM | -0.29 | **-0.93** | 0.00 | 0.00 | -0.34 |
| Inter REM | 0.22 | **0.93** | -0.01 | 0.15 | 0.42 | Inter REM | 0.00 | **0.78** | -0.17 | 0.01 | 0.02 |
| Wake min | 0.10 | 0.10 | **-0.94** | 0.13 | 0.14 | Wake min | 0.01 | 0.01 | **-0.99** | 0.01 | 0.01 |
| NREM min | 0.00 | 0.18 | **0.99** | -0.01 | -0.01 | NREM min | -0.08 | 0.06 | **0.95** | -0.13 | -0.13 |
| NREM rel Delta | 0.10 | 0.00 | 0.00 | **0.85** | 0.12 | NREM rel Delta | 0.00 | -0.11 | -0.09 | **0.75** | 0.00 |
| REM rel Theta I | 0.22 | 0.19 | 0.11 | **-0.72** | 0.14 | REM rel Theta I | 0.07 | 0.02 | 0.00 | **-0.83** | 0.01 |
| REM rel Theta II | -0.01 | -0.01 | 0.15 | **-0.77** | 0.13 | REM rel Theta II | -0.15 | -0.14 | 0.01 | **-0.86** | 0.00 |
| lat NREM | 0.31 | -0.01 | -0.01 | -0.02 | **0.93** | lat NREM | 0.09 | -0.16 | -0.20 | -0.27 | **0.60** |
| lat REM | 0.21 | 0.39 | -0.01 | 0.00 | **0.93** | lat REM | 0.02 | 0.05 | -0.20 | -0.14 | **0.67** |
| db REM | 0.25 | 0.42 | -0.01 | 0.35 | **0.77** | db REM | 0.01 | 0.01 | -0.38 | 0.02 | 0.04 |
| % of Variance | 32.4% | 18.0% | 13.9% | 12.2% | 9.8% | % of Variance | 30.6% | 16.2% | 12.4% | 10.3% | 8.1% |
| Explained by |  |  |  | TOTAL: | 86.3% | Explained by |  |  |  | TOTAL: 77.6% | |
| The Factors |  |  |  |  |  | the Factors |  |  |  |  |  |
