# Supplementary material for: Uncovering the Genetic Landscape for Multiple Sleep-Wake Traits
Source: PLoS One. 2009 Apr 10;4(4):e5161. doi: 10.1371/journal.pone.0005161 (PMC2664962; doi:10.1371/journal.pone.0005161)
Supplement: Table S3 — Expanded QTL Data (0.31 MB DOC) [file pone.0005161.s006.doc]

## **Supporting Information**

To accompany Winrow et al., 08-PONE-RA-06401R1

## **Uncovering the Genetic Landscape for Multiple Sleep-Wake Traits**

**Table S2: Bootstrap obtained 95% Confidence Intervals of Factor Analysis.**

The factor analysis was iteratively performed on 1000 bootstrapped-with-replacement samples of the original sleep-wake trait data. The upper 95% and lower 5% factor loading values from this analysis are shown with the same cells bolded as in Table 1.

Table S2: Bootstrap obtained 95% Confidence Intervals of Factor Analysis

| **Upper 95% Confidence Values** | Factor | Factor | Factor | Factor | Factor | **Lower 5% Confidence Values** | Factor | Factor | Factor | Factor | Factor |
| --- | --- | --- | --- | --- | --- | --- | --- | --- | --- | --- | --- |
|  | 1 | 2 | 3 | 4 | 5 |  | 1 | 2 | 3 | 4 | 5 |
|  | **Fragment ation** | **REM Sleep** | **State Amount** | **Power Bands** | **Latency** |  | **Fragment ation** | **REM Sleep** | **State Amount** | **Power Bands** | **Latency** |
| nb Wake | **-0.78** | -0.12 | 0.12 | 0.33 | -0.01 | nb Wake | **-0.84** | -0.28 | 0.00 | 0.18 | -0.14 |
| db Wake | **0.81** | 0.30 | -0.23 | -0.13 | 0.20 | db Wake | **0.72** | 0.16 | -0.39 | -0.31 | 0.01 |
| nb NREM | **-0.94** | -0.02 | 0.25 | 0.00 | -0.03 | nb NREM | **-0.97** | -0.12 | 0.13 | -0.08 | -0.11 |
| db NREM | **0.96** | 0.20 | 0.26 | 0.08 | 0.15 | db NREM | **0.93** | 0.09 | 0.15 | 0.00 | 0.02 |
| db TS | **0.97** | 0.14 | 0.23 | 0.08 | 0.15 | db TS | **0.94** | 0.03 | 0.12 | 0.00 | 0.02 |
| # Arousals | **-0.63** | 0.22 | 0.39 | -0.20 | -0.01 | # Arousals | **-0.74** | 0.03 | 0.17 | -0.40 | -0.18 |
| # Shifts | **-0.93** | -0.08 | 0.25 | 0.00 | -0.03 | # Shifts | **-0.96** | -0.19 | 0.13 | -0.08 | -0.12 |
| Onset REM | **0.88** | 0.23 | 0.39 | 0.11 | 0.17 | Onset REM | **0.81** | 0.10 | 0.25 | 0.00 | 0.01 |
| REM min | -0.09 | **-0.84** | -0.01 | 0.16 | 0.26 | REM min | -0.23 | **-0.95** | -0.15 | 0.01 | 0.01 |
| % REM/TS | -0.07 | **-0.76** | -0.32 | 0.17 | 0.20 | % REM/TS | -0.20 | **-0.89** | -0.54 | 0.01 | 0.01 |
| nb REM | -0.16 | **-0.84** | 0.15 | 0.10 | -0.02 | nb REM | -0.29 | **-0.93** | 0.00 | 0.00 | -0.34 |
| Inter REM | 0.22 | **0.93** | -0.01 | 0.15 | 0.42 | Inter REM | 0.00 | **0.78** | -0.17 | 0.01 | 0.02 |
| Wake min | 0.10 | 0.10 | **-0.94** | 0.13 | 0.14 | Wake min | 0.01 | 0.01 | **-0.99** | 0.01 | 0.01 |
| NREM min | 0.00 | 0.18 | **0.99** | -0.01 | -0.01 | NREM min | -0.08 | 0.06 | **0.95** | -0.13 | -0.13 |
| NREM rel Delta | 0.10 | 0.00 | 0.00 | **0.85** | 0.12 | NREM rel Delta | 0.00 | -0.11 | -0.09 | **0.75** | 0.00 |
| REM rel Theta I | 0.22 | 0.19 | 0.11 | **-0.72** | 0.14 | REM rel Theta I | 0.07 | 0.02 | 0.00 | **-0.83** | 0.01 |
| REM rel Theta II | -0.01 | -0.01 | 0.15 | **-0.77** | 0.13 | REM rel Theta II | -0.15 | -0.14 | 0.01 | **-0.86** | 0.00 |
| lat NREM | 0.31 | -0.01 | -0.01 | -0.02 | **0.93** | lat NREM | 0.09 | -0.16 | -0.20 | -0.27 | **0.60** |
| lat REM | 0.21 | 0.39 | -0.01 | 0.00 | **0.93** | lat REM | 0.02 | 0.05 | -0.20 | -0.14 | **0.67** |
| db REM | 0.25 | 0.42 | -0.01 | 0.35 | **0.77** | db REM | 0.01 | 0.01 | -0.38 | 0.02 | 0.04 |
| % of Variance | 32.4% | 18.0% | 13.9% | 12.2% | 9.8% | % of Variance | 30.6% | 16.2% | 12.4% | 10.3% | 8.1% |
| Explained by |  |  |  | TOTAL: | 86.3% | Explained by |  |  |  | TOTAL: 77.6% | |
| The Factors |  |  |  |  |  | the Factors |  |  |  |  |  |

**Table S3: Expanded QTL Data**

An expansion of the main text Table 2. QTL were detected by R package QTL as were the peak LOD scores. The QTL peak is defined as the position within the QTL with the highest LOD score. Trait type was determined by factor analysis (Table 1) and further trait description is available in Supplemental Information Part A. The start and end of the QTL was defined by the LOD score dropping to 1.0. FDR (False Discovery Rate) threshold was defined by 1000 permutations. Est is the estimated effect of the locus on the trait during the period (24-hr, Light or Dark) in the units of the trait (number, percent, etc). UL and LL define the upper and lower limit respectively of the 95% confidence interval of the estimated effect. Total is the estimated total effect of the locus on the trait over the entire 48-hr recording period. % Light (or % Dark) is the percent of the total effect that occurred during the light (or dark) period. % Total is the sum of % Light and % Dark.

**Table S.3. Expanded QTL Data**
