# Supplementary material for: Uncovering the Genetic Landscape for Multiple Sleep-Wake Traits
Source: PLoS One. 2009 Apr 10;4(4):e5161. doi: 10.1371/journal.pone.0005161 (PMC2664962; doi:10.1371/journal.pone.0005161)
Supplement: Table S4 — Correlation Tables (0.07 MB DOC) [file pone.0005161.s007.doc]

## **Supporting Information**

To accompany Winrow et al., 08-PONE-RA-06401R1

## **Uncovering the Genetic Landscape for Multiple Sleep-Wake Traits**

**Table S4: Correlation Tables**

Correlation tables comparing different light/dark periods for the 20 sleep-wake traits are shown. Traits were averaged per animal across the 14-hr light, 10-hr dark or 24-hr periods for two consecutive baseline days of recording. Pearson coefficient (R) values are shown; bold typeface indicates Bonferroni multiple-testing corrected significance (p ≤ 0.0000515). P-values are shown in the upper-right half of tables A-C, and those for table D are shown in table

**Table S4: Correlation Tables**

A. 24-hr X 24-hr

B. Light X Light

C. Dark X Dark

D. Dark X Light

E. Dark X Light p-values
