# Supplementary material for: Uncovering the Genetic Landscape for Multiple Sleep-Wake Traits
Source: PLoS One. 2009 Apr 10;4(4):e5161. doi: 10.1371/journal.pone.0005161 (PMC2664962; doi:10.1371/journal.pone.0005161)
Supplement: Table S5 — Bootstrap Results for Bayesian Network (0.03 MB DOC) [file pone.0005161.s008.doc]

## **Supporting Information**

To accompany Winrow et al., 08-PONE-RA-06401R1

## **Uncovering the Genetic Landscape for Multiple Sleep-Wake Traits**

**Table S5: Bootstrap Results for Bayesian Network**
